# ogMapper improves speed and memory for short-sequencing read mapping using a novel guided sparse genome index

**DOI:** 10.3389/fbinf.2026.1912289

**Published:** 2026-10-19

**Authors:** Victor Treviño, Eugenio Guzman-Cerezo

**Affiliations:** 1 Tecnologico de Monterrey, OriGen Project, Monterrey, Nuevo León, Mexico; 2 Tecnologico de Monterrey, School of Medicine, Monterrey, Nuevo León, Mexico; 3 Tecnologico de Monterrey, School of Engineering, Monterrey, Nuevo León, Mexico

**Keywords:** genome, genomics, mapper algorithm, reads alignment, sequencing

## Abstract

Short DNA-read sequencing has become an everyday essential tool in research and clinical setting. Read mapping is a crucial step that consumes large amounts of memory and CPU time, limiting the hardware required and ultimately restricting its applications. The memory required for the genome index can be approximated as *βG*, where *G* is the genome size and *β* is a factor that depends on the implementation. For example, *β* ≈ 10 for *bwa-mem2* or *β* ≈ 1.9 for *minimap2*. Here, an indexing strategy is presented in which *β* can be even less than 1, commonly around 0.5. The indexing strategy relies on “guides,” a deterministic, sequence-based pattern for key selection that allows the search to directly access key occurrences. The C++ implementation, available on GitHub as *ogMapper* (oriGen mapper), is faster than current methods on the T2T human genome, with a negligible reduction in the percentage of reads mapped, and shows memory usage less than 3 GiB even with multiple mapping threads. *ogMapper* can be used for DNA-Seq reads and has been tested on macOS X, Linux, and Android systems. The *ogMapper* code is available on GitHub at https://github.com/vtrevino/ogmapper, including installation, compilation, and running instructions. Using sequencing data from simulations and well-characterized samples from Genome in a Bottle, we demonstrate the capabilities of *ogMapper* by comparing it with other short-read mappers and analyzing possible downstream effects using three variant callers.

## Introduction

DNA and RNA sequencing have become essential tools in research and clinics where millions of short (≈ 150 bp) or long reads (> 1,000 bp) are produced every day around the world, from large genomics centers to small clinics and laboratories (Polonis et al., 2025), generating thousands of petabytes per year (Davis-Turak et al., 2017). Mapping is a critical step designed to find the position where each read belongs in a reference genome (Canzar and Salzberg, 2017). This process is needed for the subsequent identification of genomic alterations (Betschart et al., 2022). For mapping, an index of the genome must be built and is commonly loaded into memory, which is critical for speeding up the searches and alignments of millions of reads. Recently, indexes have drawn attention because they generate performance bottlenecks (Jung and Han, 2022). However, recent proposals focus on improving index access, with little attention being given to reducing index size. For instance, *bwa-meme* improves performance over *bwa-mem2* but recommends more than 140 GiB of RAM (Jung and Han, 2022). Currently, the index size and the memory used can be modeled as a function of the genome size as f(G) = β*G, where *G* is the genome size and *β > 1*, typically depending on the implementation. For example, for the telomere-to-telomere human genome assembly (T2Tv2), *β* ≈ 10 or more in the most popular mapper, *bwa-mem2*, which uses an FM-index, a compressed form of full-text substrings from the Burrows-Wheeler transform (Md et al., 2019), while *β* ≈ 1.9 in *minimap2*, which uses substring minimizers (Li, 2018).

## Concept and implementation

In *minimap2*, where *β* is around 2, the reduction in index size is achieved because the *k-mer* minimizer is likely to be the same across neighboring windows of length *w* (Figure 1). This corresponds to the concept of “sparse-indexing,” wherein not all possible *k-mers* are indexed but rather only those that serve as minimizers within windows. Minimizers belong to a string theory of *syncmers* (*s-mers*) (Edgar, 2021), which may further improve *k-mer*-based operations (Edgar, 2021; Shaw and Yu, 2022) while conserving the selection of specific substrings and, therefore, the use of sparse indexing. Examples of *k-* and *s-mers* from a sequence are shown in Figure 1. Instead of using k-mers or syncmers, we were inspired by recognition sites from endonucleases and CRISPR technology to detect recurrent patterns in DNA sequences that can be used to generate sparse indexes. The sequence pattern selector is referred to here as a “guide” sequence that indicates the indexing site.

**FIGURE 1:**
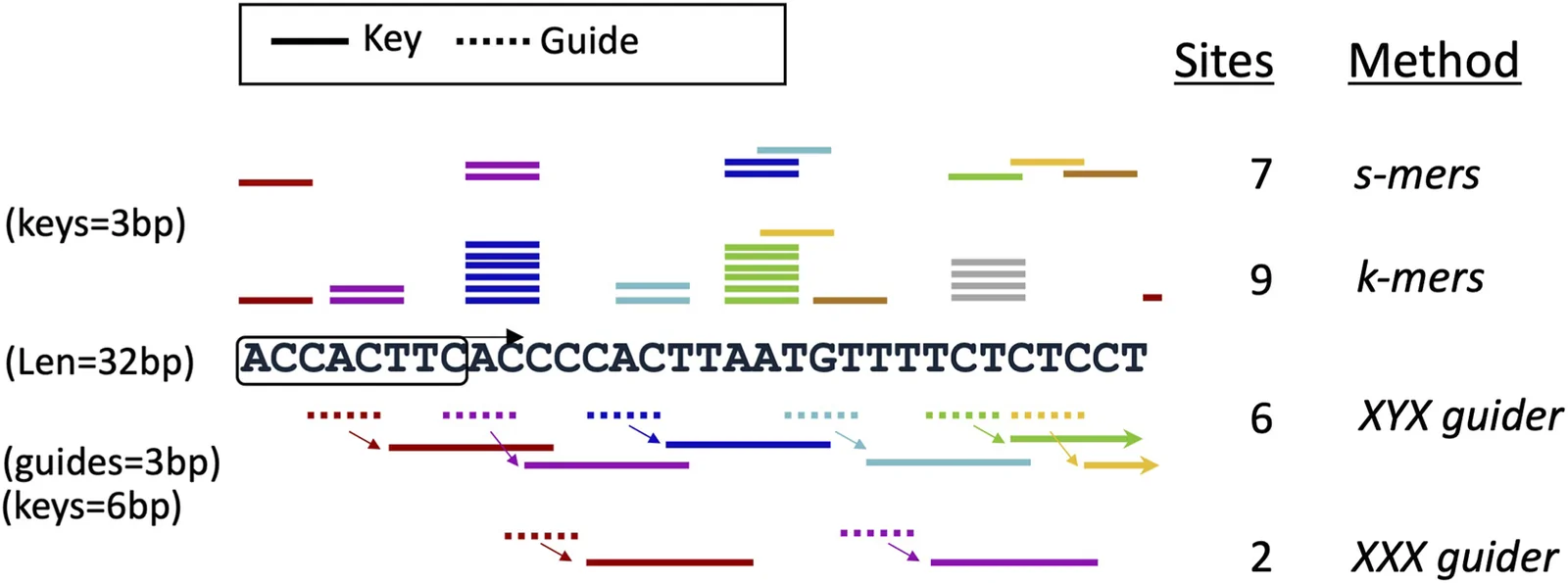
Comparison of sites selected for indexing by different methods. The syncmers’ methods (s- and k-mers) select indexing sites by analyzing sub-k-mers within sliding windows (boxes), typically using the minimal lexicographical k-mer or any other function. The window is size = 8 bp (6 k-mers) and k = 3. In guided sparse indexing, a guiding pattern is searched for in the sequence, marking positions to be indexed. The indexed key is a nearby sequence rather than the guide itself, which is marked by dotted lines. The key length is intended to be larger with respect to the guide. Here, one guide is the pattern XYX, where X is any bp and Y is a different bp from X. Thus, the pattern will match only ACA, AGA, ATA, CAC, CGC, CTC, GAG, GCG, GTG, TAT, TCT, and TGT. Any other triplet does not match. The second guide pattern is XXX, where triplets AAA, CCC, GGG, and TTT will match, while the other 60 combinations do not. In the XYX example, the result is 2 incomplete and 4 complete guide–key pairs from 24 possible guide positions in a 32 bp sequence (32 + 1 − 3 guide − 6 key). Only complete keys are utilized.

In guided sparse indexing, the guide is a sequence pattern used to select the *key* to be indexed (Figure 2a). For example, in Figure 2a, the key (continuous line) is extracted immediately following the guide (dotted line). This helps distinguish between two elements: “where to index” provided by the guide, and “what to index” provided by the key. Interestingly, in guided sparse indexing, the key also carries information from the guide as only occurrences of the key that are preceded by a guide are indexed. Overlapping guides are not allowed to avoid over-indexing in low-complexity regions (such as GGGGGG … or TCTCTC….). Since the key sequence is algorithmically independent of the guide, in sufficiently large genomes, the frequency of key sequences should be widely distributed across all possible key combinations. This feature may reduce the search space and perhaps accelerate the mapping (Supplementary Figure S1). Theoretically, less frequent guides result in fewer keys and thus smaller indexes. Therefore, the ratio of keys to DNA positions (key ratio) is used as a proxy measure of index size, which can then be translated to *β* depending on the implementation. Notably, very rare guides would increase the likelihood of larger regions lacking the guiding pattern, thus also increasing the number of unmappable regions. This issue is avoided by introducing an exception rule: “index the current location if no guiding pattern is found within *M* bp”. Too many exceptions are also undesirable, so when the rule applies, the current position is indexed, and *S* nucleotides are skipped, where *S = M/ guide length*. For genome indexing (Figure 2b), a scan is performed to search for guides and extract keys to build the index vectors. Succinctly, the index size is determined by the total number of guides plus the exceptions. Subsequently, for read mapping (Figure 2c), the same guide–key process is applied, with the keys serving as seeds for searching the indexed genome. Given that reads can contain sequencing errors or individual variants, it is crucial for reads to contain several keys to improve mapping efficiency.

**FIGURE 2:**
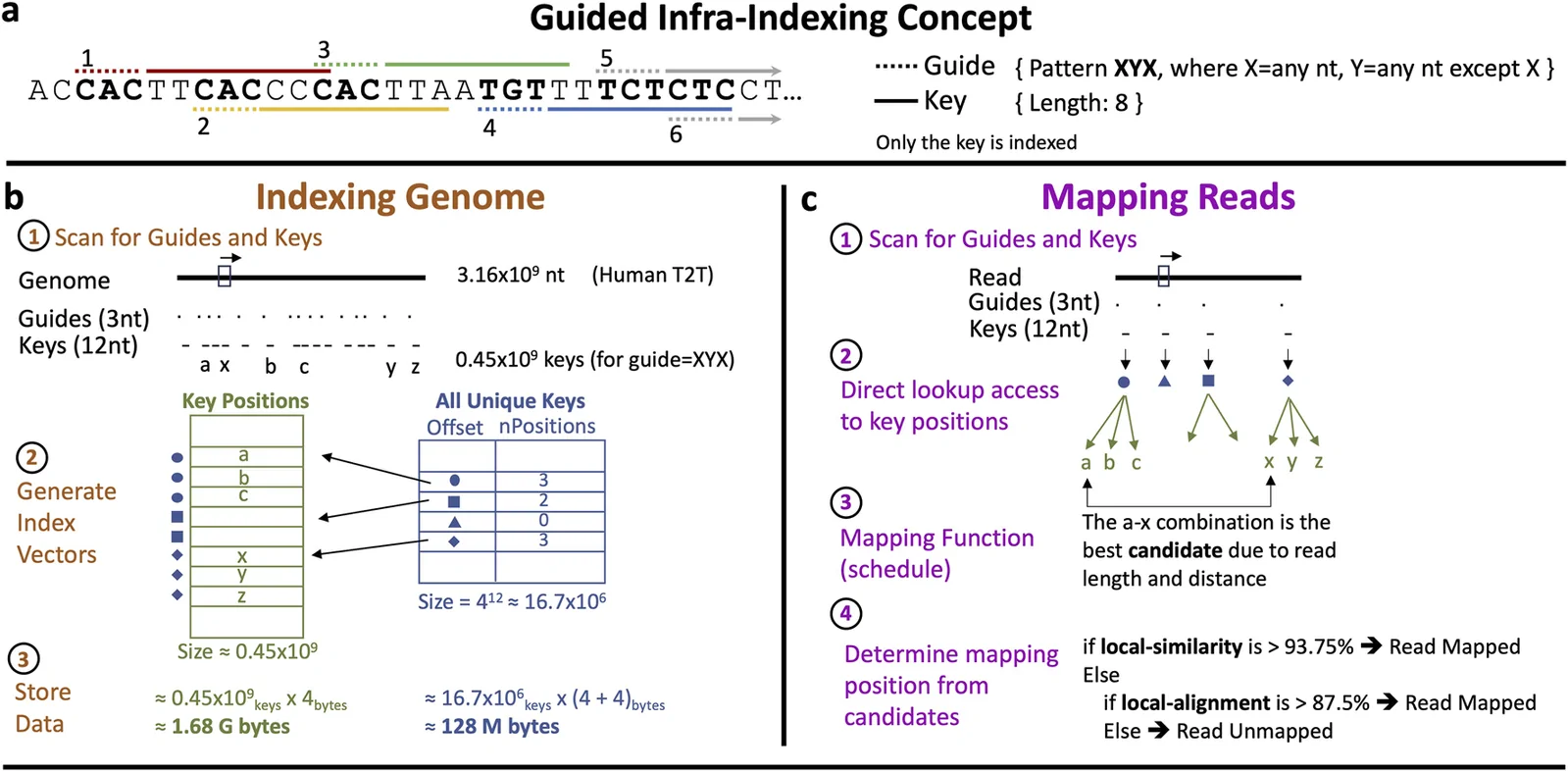
Guided infra-indexing concept and implementation. **(a)** A guiding pattern is searched for in the DNA sequence, marking key positions to be indexed. The indexed key (continuous line) is a nearby sequence rather than the guide itself (dotted lines). The key length is intended to be larger than the guide. Here, the guide is the pattern XYX, where X is any bp and Y is a different bp from X. Thus, the pattern will match only ACA, AGA, ATA, CAC, CGC, CTC, GAG, GCG, GTG, TAT, TCT, and TGT. Any other triplet does not match. The result is 4 complete guide–key pairs from 22 possible guide positions in the 32 bp sequence shown (32 − 3 − 8 + 1). **(b)** To index a genome, every chromosome is scanned for the guide pattern to register all guided keys. Two vectors are generated and stored. **(c)** To map reads, their sequences are scanned for the guide pattern, and the corresponding key positions are accessed by the mapping functions. Several functions could be devised. The figure exemplifies a function that looks for key intersecting positions at the ends. Other functions are explained in the GitHub repository. Local similarity is assessed before alignment, assuming that most reads do not contain indels, where a quick direct comparison is highly efficient. Alignment is performed using the WFA2 library, as described in the Methods section.

To explore diverse guiding patterns, the expected and observed key ratios of different common patterns that can serve as *guides* spanning 2 to 4 nucleotides in the T2T-CHM13-v2 human genome are compared in Figure 3a and Supplementary Table S1 (using *M = 24*). As can be noted, the observed ratios of the target guide patterns were close to the expected ratios. Nevertheless, the level of exceptions is guide-specific and is higher in less frequent guiding patterns, such as those with key ratios around 0.05. The largest index was generated by the simpler 2-repetition guiding pattern (XX = AA, CC, GG, TT), showing an observed key ratio of 0.22 and an index size of 3 GiB, which represents a *β = 0.92*, still below 1. The XYX pattern, with a key ratio of 0.142, generated an index of only 2 GiB (*β = 0.58*) and produces, on average, around 13 keys in 150-bp reads (see Methods), which can be highly attractive in practice. Nevertheless, the lowest index size tested was XXX + of 1.4 GiB (*β = 0.42*), generating 202.1 M occurrences of the guiding pattern plus 90.3 M key occurrences due to the exception rule.

**FIGURE 3:**
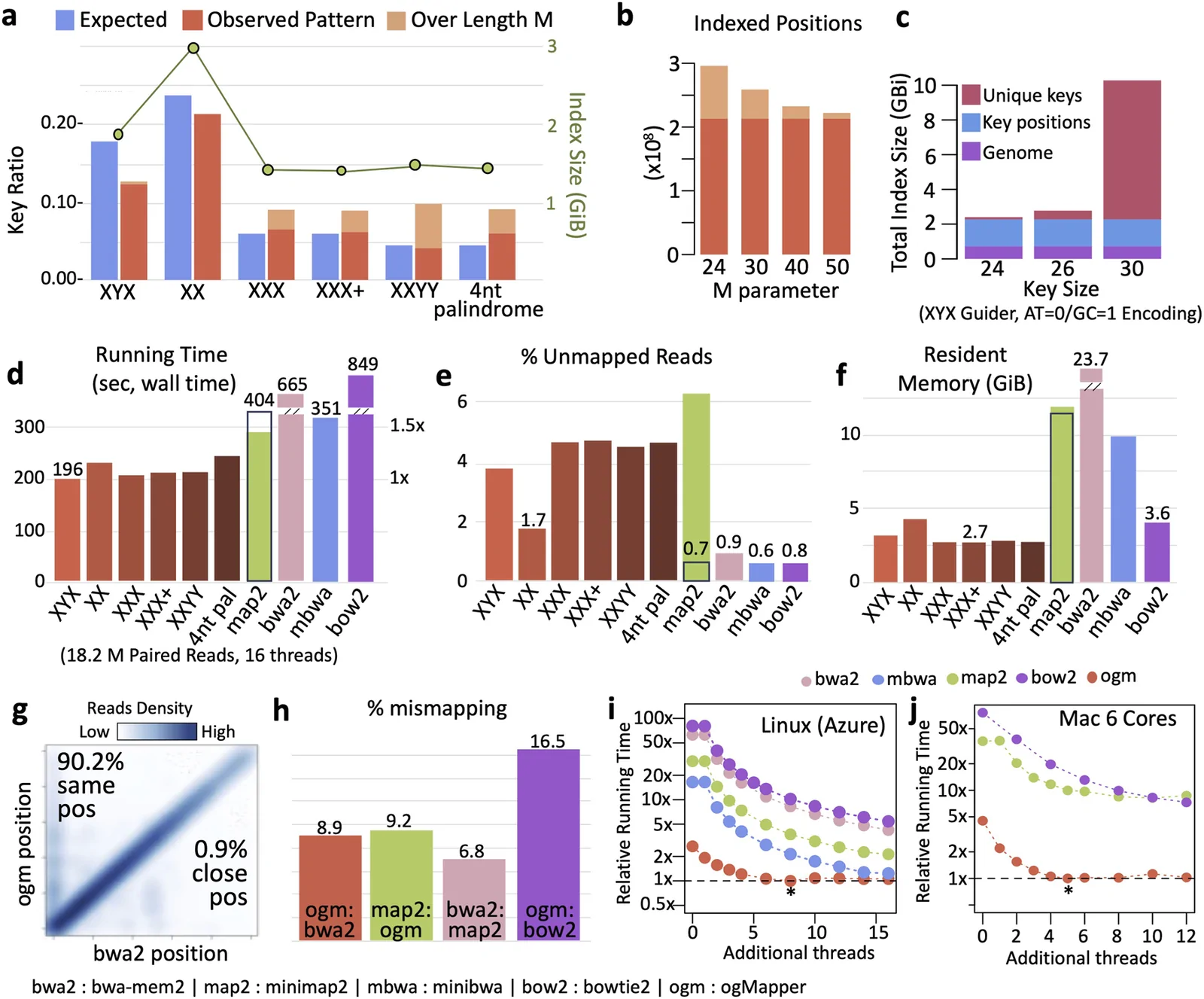
Results of the ogMapper implementation. **(a)** Expected versus observed frequencies of diverse guiding patterns. The key ratio is defined as the ratio of the number of keys that would be indexed to all possible DNA positions. Over length M refers to the number of observed exception rules at M = 24. The index size in GiB is also shown (not including the packed genome). **(b)** Indexed positions as a function of the exception rule M value for the XXX guide. **(c)** Index size by varying key size. **(d)** Running time comparison of several guiding patterns in *ogMapper* (ogm) compared to *minimap2* (map2), *bwa-mem2* (bwa2), *minibwa* (mbwa), and *bowtie2* (bow). Only mapping time is measured (indexing time is not included). **(e)** Percent of unmapped reads across guides and mapping tools. **(f)** Observed resident memory consumed by tools and guide runs as reported by *time -al* in Linux. The black empty bar in *minimap2* (map2) in panels **(d,e,f)** corresponds to runs using the short reads preset (-x sr). The black empty box in *ogMapper* XYX corresponds to runs using the option -s x4sXS. **(g)** Comparison of read-mapping positions between *ogMapper* and *bwa-mem2*. **(h)** Overall mismapping among tools. **(i,j)** Relative running time with additional threads in Linux and macOS. “*” marks the run used as the reference (vertical axis in logarithmic scale).

The distribution of genome positions per key is crucial for mapping efficiency. In *minimap2*, the minimizers tend to be more frequent for low lexicographical values (or low-coded) because they are selectively preferred (e.g., AAA over AAC if lexicographical or 00* over 11* keys if coded). Instead, in our approach, the *guide:key* combination represents real genome frequency. The observed distribution shows that most *guide: key* combinations are present at fewer than 100 genome positions (Supplementary Figure S3a). Increasing the key size also increases the index size and mapping speed but decreases the percentage of mapped reads and increases the number of empty keys (Supplementary Figure S4). The index size is slightly dependent on the *M* parameter when the number of indexed positions due to the exception rule is considerable. For example, the XXX guide decreases by 210 MiB (≈ 15%) when *M = 50* compared with *M = 24* (Figure 3b).

The index size also depends on how the DNA sequence is encoded into a key. A plain 4-bp representation uses 2 bits per bp (e.g., A = 00, C = 01, G = 10, T/U = 11, N or any other character is encoded as A). We tested whether other DNA encodings could improve the indexing–mapping problem, particularly those that use less than 2 bits per bp. An encoding that uses 1 bit per bp (A = 1, T = 1, C = 0, G = 0) showed a broader distribution of keys (Supplementary Figure S3b). For instance, plain encoding generates ≈2M empty keys (11% with no genome positions) for 12-bp keys using 24 bits, while the above 1-bit encoding generates fewer than 200K empty keys (1%) for 24-bp keys also using 24 bits. Unless otherwise stated, experiments were performed using the above 1-bit per bp encoding; other encodings are shown in Supplementary Table S2.

The index size is also affected by the key length. In our implementation, we avoided the binary search employed to look for a key and opted for direct access to key positions. So, we store the starting position and length of all possible keys (Figure 2b; Supplementary Figure S1). To show the effect of the key length, we compared the index size using incremental key sizes (Figure 3c). At a key size of 30, which uses 30 bits in the encoding used, the index requires 8 GiB to store the starting position and length of each key, and 80% of them are empty (864846671 of 1073741824). In contrast, at a key size of 24 bits, only 128 MiB are needed, and only 1.6% of them are empty (263784 of 16777216).

These results show that the guiding concept is flexible and influences index size.

The mapping efficiency for diverse guides using the T2Tv2 human genome as the reference was compared using 18.2 M paired-end reads from Genome in a Bottle data on a Linux machine in the Azure cloud (D32adsv5 model). For *minimap2* and *bwa-mem2*, 16 threads (-t parameter) were used, while only 15 additional threads were used in *ogMapper* because the current implementation may use the root thread also for mapping when working thread queues are full (although the root thread was used on average ≈ 5% of the time for mapping). Overall, *ogMapper* was between 1.45 and 1.25 times faster than *minimap2*, and between 3.39 and 2.77 times faster than *bwa-mem2* (Figure 3d). Given that in our approach fewer genome positions are indexed, the percentage of mapped reads is expected to decrease. However, most guides showed around 4% unmapped reads (Figure 3e). Interestingly, the XX guide reached 1.7% unmapped, which was the closest to the standard tool, *bwa-mem2*, at 0.9%. This is explained by the increased indexed positions covered in the XX guide. Nevertheless, a fraction of the unmapped reads can also be ascribed to the mapping functions (schedule functions in *ogMapper*) because adding the options XS to -s (using -s x4sXS) decreases the proportion of the unmapped reads to 1.1% for the XYX guide without substantially increasing the running time. In *ogMapper*, the resident memory used is almost equivalent to the index size plus the packed genome size of around 800 MiB for T2Tv2 (included in the index file), which was lowest in the XXX + guide, reaching only 2.7 GiB of RAM and up to 4.3 GiB for the XX guide (Figure 3f). This represents up to 4.4 times less memory than *minimap2* and 8.7 times less memory requirement than *bwa-mem2*.

To further demonstrate the memory efficiency, we also tested *ogMapper* on an Android mobile phone with 6 GiB of RAM and 8 cores (OnePlus A5010). Indexing the human hg38 took 8 min and 25 s, while mapping 4M paired-end reads took 2 min and 16 s using 4 additional threads. Both processes used 2.7 GiB of RAM. Instructions are provided in the GitHub repository.

Performance was also compared according to the number of threads. Because indexing is needed only once per genome, we did not compare the indexing times. All mappers became faster as the number of threads increased, but only up to a limit, around 5 for *ogMapper* and higher for *minimap2* and *bwa-mem2* (Figures 3h,i). *ogMapper* was faster than *minimap2* and *bwa-mem2* and the recently released *minibwa* regardless of the number of threads. *Bowtie2* was the slowest in our runs.

To assess mapping precision, the mapping position was compared among mappers (*ogMapper*, *bwa-mem2*, *minimap2*, *minibwa* (Li and Homer, 2026), and *bowtie2* (Langmead and Salzberg, 2012)). We used the runs with the lowest unmapped reads for the comparison (XX for *ogMapper* and *-x sr* for *minimap2*). A mismatch was determined when the difference in genome position was greater than 150 bp to account for possible displacements due to soft-clipping operations reflected in the CIGAR field. Up to 90.2% of the reads mapped to exactly the same position with *ogMapper* and *bwa-mem2* (Figure 3g), while only 0.9% of the reads mapped to positions with a difference of less than 150 bp, and approximately 60% of these mapped within 20 bp, probably due to differences in the CIGAR field. The overall mismapping was 8.9% between *ogMapper* and *bwa-mem2*, which is higher but comparable to that between *minimap2* and *bwa-mem2*, which reached 6.8% (Figure 3h). Up to 66% of the mismapped reads between *ogMapper* and *bwa-mem2* were also mismapped between *minimap2* and *bwa-mem2*, suggesting that these are reads that are difficult to map. We also tested *ogMapper* using the XYX guide with 1M simulated random reads from the source genome; 94% of the reads were mapped, and 88.5% mapped exactly to the extracted position, confirming the overall performance. The highest difference observed was when compared with *bowtie2*.

To evaluate the possible downstream effects of the output generated by *ogMapper* in common pipelines, we first estimated the resulting coverage depth, with the maximum reached by *bwa-mem2* at 21.7x compared with 20.9x for *ogMapper* reaching, indicating a relative effectiveness of 96%, presumably attributable to the 4% of unmapped reads (Figure 4a). We then compared the total, shared, and exclusive variants called after mapping across aligner tools (see Methods). Despite the 4% of unmapped reads, the number of high-quality variants called by GraphTyper2 (Eggertsson et al., 2019) from the *ogMapper* output was highly competitive compared with other aligners, and was even higher than that of *bowtie2* (Figure 4b). Moreover, approximately 89% of the 3.7 M variants called by *ogMapper* were also called by *bwa-mem2*, *minimap2*, and *minibwa* (Figure 4c). The overlap decreased to 80% compared with *bowtie2*, partially due in part to its lower number of variants called. These results support the effectiveness of *ogMapper* for downstream applications.

**FIGURE 4:**
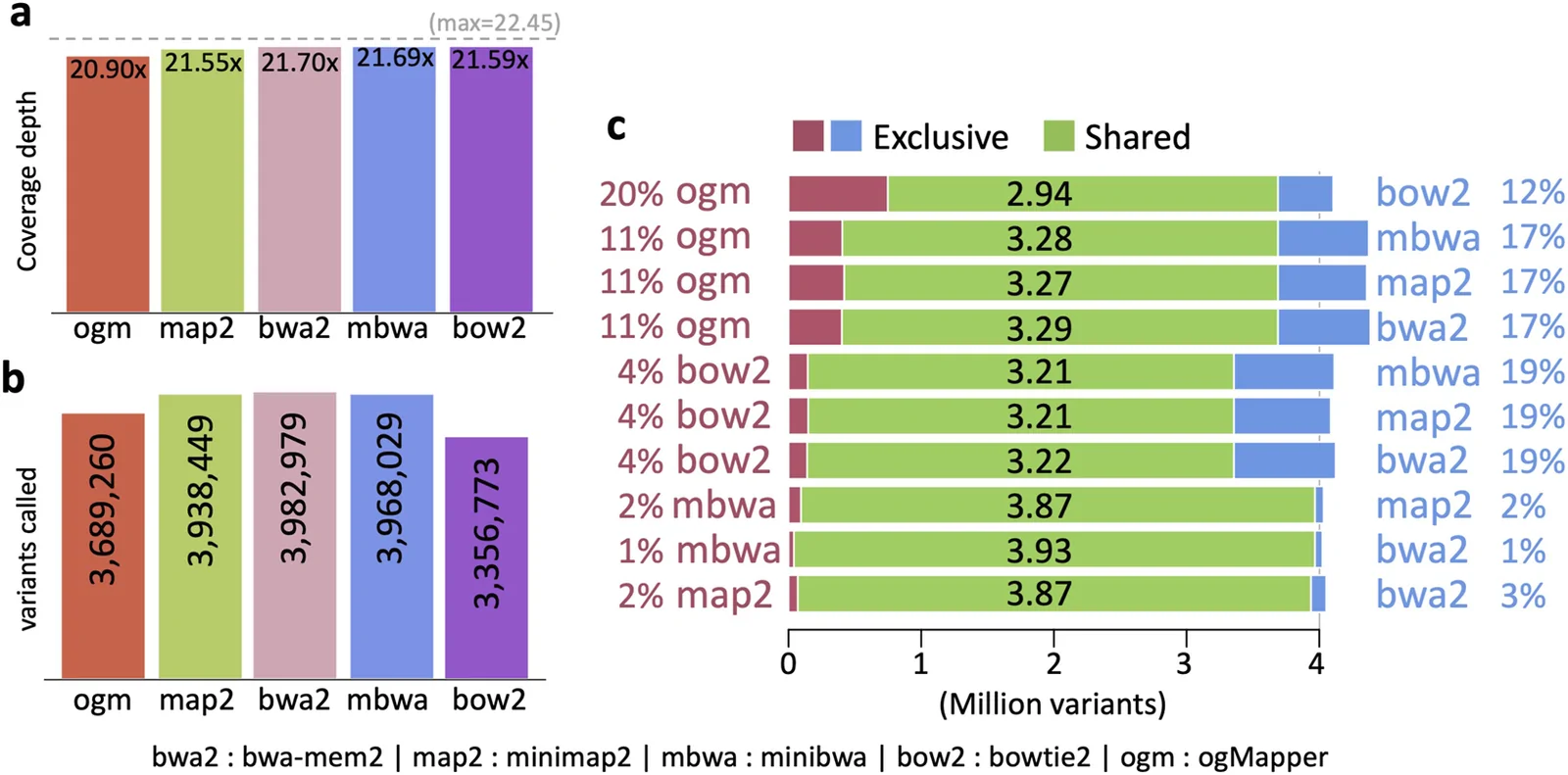
Comparison of variants called by GraphTyper2 across aligners. **(a)** Estimated coverage depth from 232 M reads. The theoretical maximum is estimated supposing non-overlapping 150bpx2 reads and 3.1 Gbp of the genome. **(b)** Total number of variants per tool after filtering. **(c)** Each bar represents the proportion of variants exclusive to the tool at left in red, shared between both tools in green, and exclusive to the tool at right in blue. The percentages at left and right represent the variants that were exclusive to the corresponding tool. The number within the green bar corresponds to the total number of shared variants in millions.

We also made a comparison between different variant callers, Haplotype Caller (Poplin et al., 2017), GraphTyper2 (Eggertsson et al., 2019), and mPileUp (Danecek et al., 2021) (Figure 5a). Haplotype Caller was the top variant detector, identifying 5.5 million from the *ogMapper* output, followed by GraphTyper2, which detected 3.7 million, and finally mPileUp, which detected only 1.9 million. The overlap with *bwa-mem2* was highest using Haplotype Caller, followed by GraphTyper2; both algorithms realign reads to detect variants. We noted that approximately 53% of the variants called only by *ogMapper* were placed in centromeres irrespective of the variant calling algorithm (Figures 5b,c), which are well-known difficult-to-map regions (Dwarshuis et al., 2024). For *bwa-mem2*, the overall proportion of variants within centromere regions varied with the variant calling algorithm, ranging from 11% to 46% (Figure 5c). In contrast, the proportion of variants in exonic regions seemed proportional to that in the overall set of variants (Figure 5d).

**FIGURE 5:**
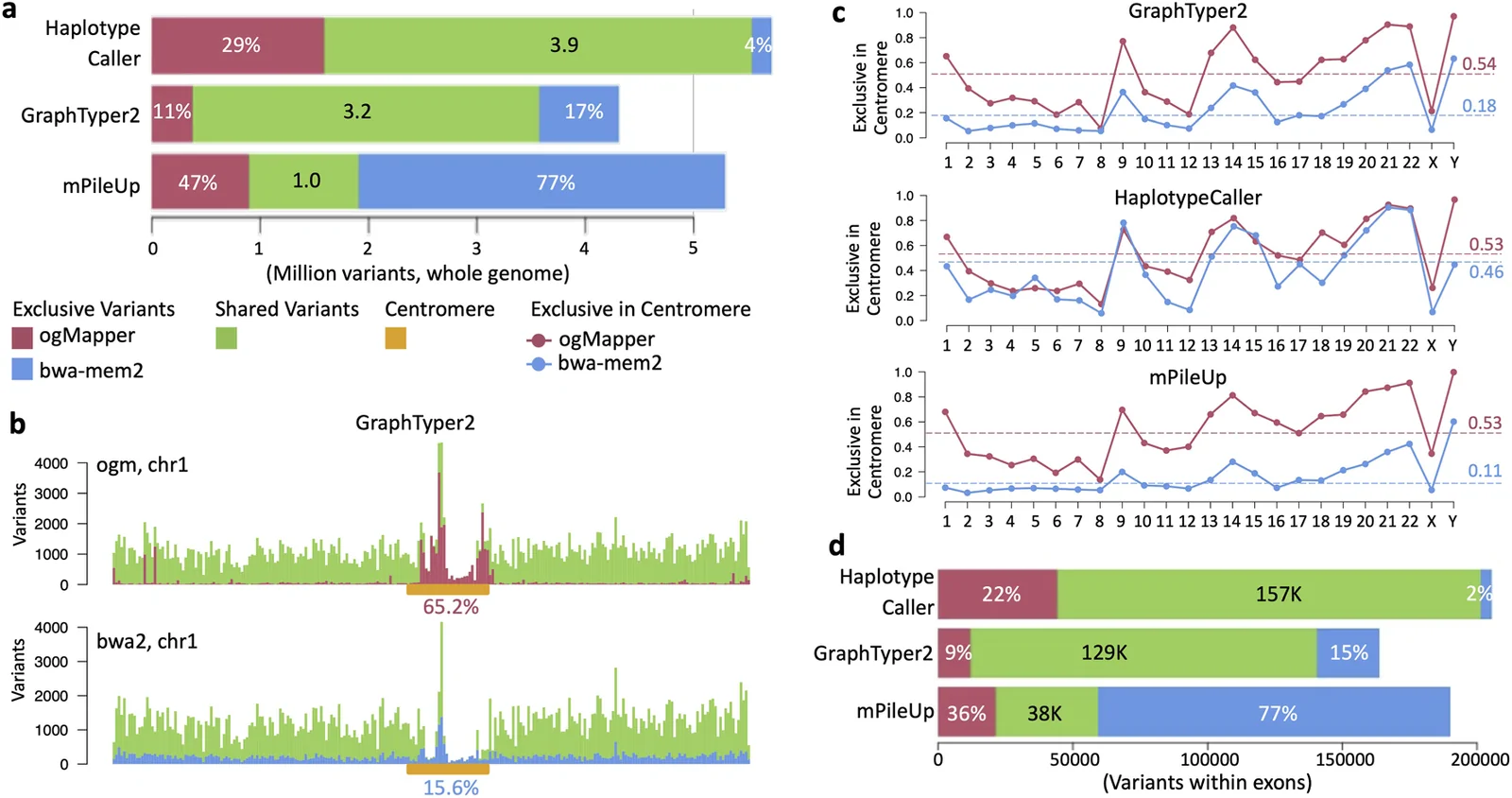
Comparison of variants called by three algorithms. **(a)** Proportion of variants shared (in green) or exclusive for *ogMapper* (red) or *bwa-mem2* (blue). **(b)** Number of variants along chromosome 1. Each bar represents 1 Mbp. The centromere region is marked in orange. The percentage below the centromere represents the number of exclusive variants within the centromere. The complete figures for all 24 chromosomes and variant callers are shown in Supplementary Figures S5–S7. **(c)** Fraction of variants placed at centromeres for each chromosome across variant callers. **(d)** Proportion of variants placed at exons. **(a,d)** The percentages on the left and right represent the variants that were exclusive to the corresponding tool, while the number within the green bar represents the total number of shared variants in millions.

To assess specificity in the estimated variants generated downstream of the use of *ogMapper*, we used the sample HG002 from the Genome in a Bottle (GIB) project. This sample represents the son of a family trio (mother, father, and son), which is a well-characterized sample set from the GIB project. We used more than 800M reads (∼80x, see Methods) and the benchmark set of variants that had been carefully called using their pipeline (Hansen et al., 2026). In addition, GIB provides a BED file containing regions where the variants are well characterized. We compared the variants called by GIB with those resulting from HaplotypeCaller using either *ogMapper* or *bwa-mem2* as a comparison. The results are shown in Figure 6. When comparing all variants or only those within the BED, *ogMapper* found more variants than *bwa-mem2* (4.91 million vs. 4.47 million in all and 4.11 M vs. 4.09 M in the BED). Nevertheless, *ogMapper* generated more exclusive variants not reported by GIB than *bwa-mem2*. Within these exclusive variants, we noted that only a minority were also located within the centromeres (Figure 6b). Thus, as a practical attempt to reduce these exclusive variants, we filtered out those whose quality may be questionable by removing variants with a low read depth of the alternative allele (AD1/(AD0+1) < 0.25). As a result, the majority of variants shared with GIB were retained, while the number of exclusive variants was greatly reduced to an acceptable level (Figure 6a, filtered bars).

**FIGURE 6:**
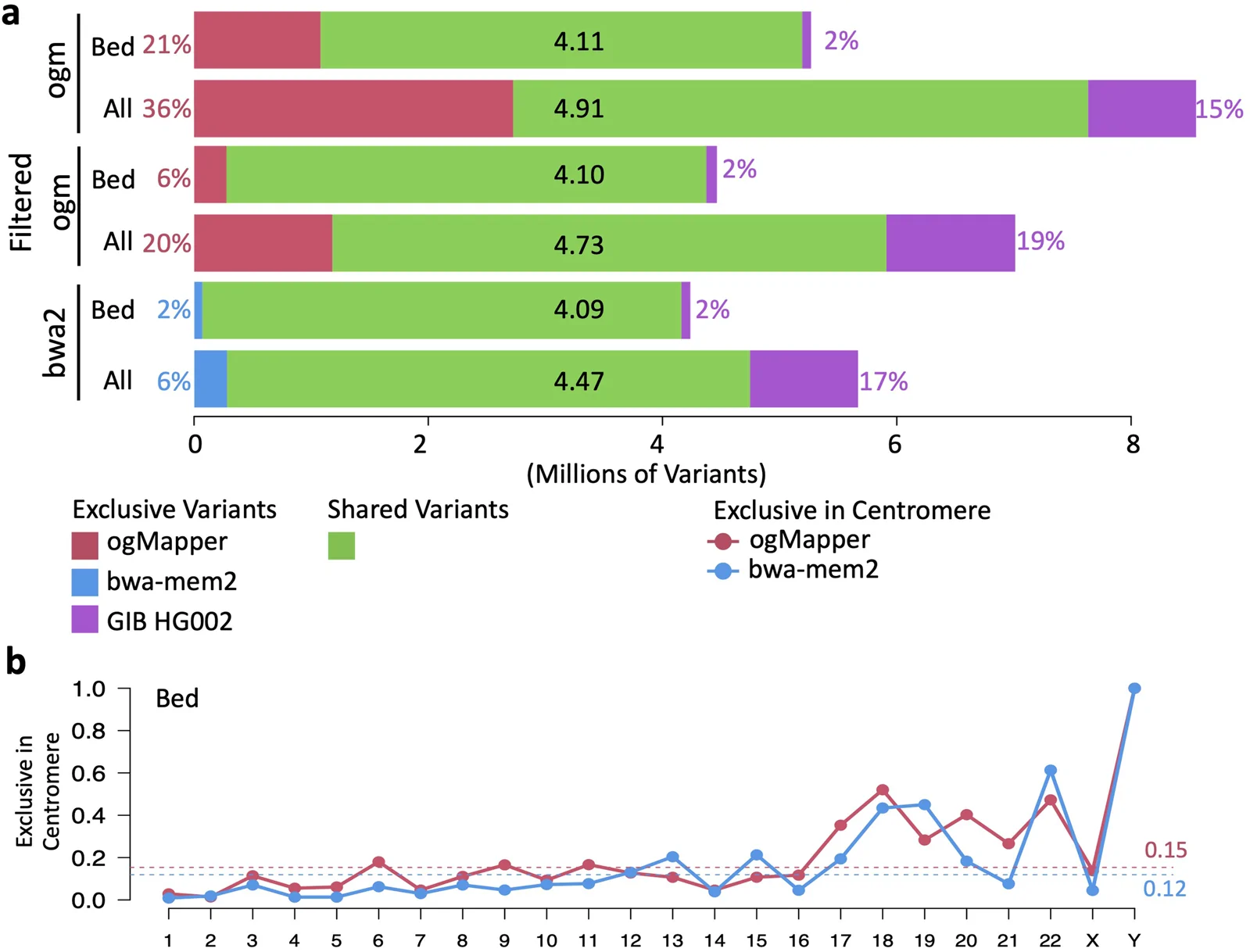
Comparison of variants called to HG002 sample from the Genome in a Bottle project. **(a)** Shared (in green) and exclusive (in red, blue, or purple) variants. The numbers within bars correspond to millions of shared variants. The percentages correspond to exclusive variants. The “All” bars represent the comparison using all variants. The “Bed” bars represent the comparison using the BED file recommended by GIB. The filtered set for ogMapper refers to variants filtered by genotype quality, with those with depth ratio AD1/(AD0+1) < 0.25 removed before comparison. AD1 refers to alternative allele read depth, while AD0 refers to the reference allele depth. **(b)** The fraction of variants placed at centromeres for each chromosome, using the variants filtered from the BED file.

## Discussion

In this manuscript, we focused on decreasing index sizes from DNA sequences, which possible improve speed. This is related to the philosophical question of whether all “k-mers” from a DNA sequence should be indexed in order to solve practical problems such as DNA mapping. Instead of indexing all substrings, *minimap2* uses k-mer minimizers, which are part of the syncmers theory. One natural thought is whether our proposal, “guided sparse indexing,” is an instance of k-mers or syncmers. We believe it does not. In guided sparse indexing, the window exists only for the exception rule, to avoid indexing large fragments of DNA, rather than for selecting a substring from the window. Even in the exception rule, the substring is chosen entirely based on its position rather than on any lexicographic criterion. Our idea is to control how many DNA fragments are indexed from a whole DNA sequence; guiding patterns provide this control because the frequency of guiding sequences in random DNA sequences can be intuitively approximated by dividing the number of positive sequences by 4^G^, where G is the length of the guide. A clear difference is also the distinction between two sequences: the guide and the key; keys can be chosen from any position relative to the guide (here, we used a neighboring key). Because several non-overlapping guides can be found in a window of DNA, guided infra-indexing may select several keys within a fragment (or window), which is not allowed in k-mers and syncmers. Another difference is the existence of ties; when the same k-mer is present at different positions, the implementation needs to decide which k-mer to index. In guiding, there are no such ties. In summary, guiding sparse indexing is a different framework.

Regardless of our specific implementation, we believe that our key-guiding approach represents a fundamentally different strategy from those used by conventional read mappers. We have demonstrated that an implementation of this concept can be both fast and memory-efficient. We consider this conceptual contribution to be the most important aspect of our work.

We successfully tested our implementation on an Android device. This proof-of-concept test demonstrates that read mapping can be performed on cheaper or simpler devices, potentially enabling novel applications.

One limitation of our approach is the slight increase in the number of unmapped reads. We will work to address this issue in future versions. Meanwhile, there are options that can be used to reduce the number of unmapped reads. For instance, adding XS to schedule functions (-s x4sXS) or even expanding to X5SZ (-s x4sX5SZ) substantially decreases the number of unmapped reads at the cost of a slight increase in running time. Moreover, the tool can still be used by coupling it with another mapper to process the unmapped reads (an option is provided to facilitate this by batching the unmapped reads into different files) when greater speed is needed and a slight increase in unmapped reads is not a concern. Other option is using low-unmapping schemes such as the XX guide.

Another limitation was observed during the analysis of the HG002 sample from GIB, in which an elevated number of false-positive variants were generated. We partially addressed this by filtering out low-quality variant calls. Nevertheless, we will work to avoid the generation of these variants. We believe that tuning certain parameters, such as the minimum similarity, may help.

Regarding other tools, we observed that *bowtie2* had a low memory footprint; nevertheless, it was the slowest. While we were submitting this manuscript, *minibwa* was released and recommended by a reviewer. *minibwa* combines approaches from *minimap2* and *bwa-mem2*. Although it was slower than *ogMapper*, it showed a notable improvement, suggesting that further innovations may contribute to improving the efficiency of read mapping.

## Conclusion

Our results demonstrate that the short-read DNA mapping problem can be improved using unexplored strategies such as guided keys, key encodings, and schedule functions for mapping. As demonstrated here, guided sparse-indexing shows an excellent computational tradeoff between speed, memory, and DNA mapping efficiency, opening new avenues for DNA applications and further theoretical and experimental analyses.

## Methods

For *minimap2*, the default *k* value (k=15) was used, generating a 7.03 GiB in index size, including around 0.7 GiB of the packed genome. As a comparison, the index file decreases to 5.8 GiB for *k=12* and increases to 8.7 GiB for *k=18*. The mapping command followed was “*minimap2 -t <n> -a -o mm.sam chm13v2.mmi U0a_R1.fq.gz U0a_R2.fq.gz*”. We did not use presets for short reads (-x sr) because we preferred faster runs, and we noted a detrimental effect in run-time when using *-x sr* (from 10% to 5x depending on the number of threads), although we also observed better memory usage (from 14 GiB to 12 GiB) and more mapped reads (Figures 3d,e). For *bwa-mem2*, the default settings for index generation (k=19) and the avx2 compilation were used. The mapping command followed was “*bwa-mem2 mem -t <n> -K 10000000 CHM13v2.fna.gz U0a_R1.fq.gz U0a_R2.fq.gz > bw.sam*”. For bowtie2, the prototype command used was “*bowtie2 -p <n> -x chm13v2 -1 U0a_R1.fq.gz -2 U0a_R2.fq.gz -S u0a-bow.sam*”. For minibwa the prototype command used was “”. For *ogMapper*, the following command “*ogmapper map -s x4s -t <n> -p 1 -i chm13v2-XXX-BW.ogi -o og.sam -2 U0a_R1.fq.gz U0a_R2.fq.gz*” was used. For paired reads mapping, *ogMapper* uses the algorithm shown in Supplementary Figure S8. *ogMapper* has options for guides and encodings, which are explicitly shown when running without arguments and described in the repository. For indexing in *ogMapper*, various options were used, but a representative for the XXX pattern is “*ogmapper index -k 24 -g TupleGuider:XXX-Tuple-og.txt -e BitwiseAT1GC0Encoding -o chm13v2-XXX-BW chm13v2.0.fa.gz*”. This indexing command takes around 230 s (< 4 min) in Azure Linux and uses 2.8 GiB of peak resident memory and 239 s (4 min) in a 2019 Intel/Mac using 2.9 GiB of memory. Indexing is forward only in the current implementation, while reads are mapped in both directions. For comparisons or different guide patterns, different tuple files (*-Tuple-*.txt, which are included in the GitHub repository) were used. For guides of variable size (e.g. XXX+), we implemented a simplified version of *State Machines*. The input is a text file containing the states and the transitions for any valid nucleotide. An example is provided for the XXX+ guide. To use the *StateMachine* guiding for the XXX+ pattern, the command was adapted to “*-g StateMachineGuider:XXXpRep-StateMachine-og.txt”* and other encodings imply changing the command to “*-e PlainEncoding -k 12*”. Other options are also available. For simulated reads, we used the mason package (https://github.com/seqan/mason) using *mason_simulator*.

For mapping in *ogMapper*, several functions were implemented that can be “scheduled” by the “-s” parameter, whose default is “x4s”. This instructs *ogMapper* to try first the function “*x”*, then if unable to map, try the function “*4”*, and then, if unable to map, try the function “*s”*. If all functions are unable to map, the read is declared unmapped. In this way, the user can control which function starts and follows. The letters correspond to a shortcut for specific mapping functions. The functions are described and listed in the GitHub repository. Briefly, suppose that a read has *k* keys, and key *i* is any of these keys; “x” corresponds to a function comparing the read and the sequences pointed by all genome positions for key *i*, keeping those positions with top matches. If any surpass a threshold, the read is considered mapped. Keys of a lower number of genome positions are analyzed first. Function “4” divides the read keys into left and right and tries to find genome positions that intersect the left and the right, considering the distance between them and a small indel. Then, top matches are aligned, and if passing a threshold, the read is considered mapped. Function “s” processes the read similar to “4” but for two non-overlapping keys. In all functions, a key filtering is done previously. See the repository for details.

### Estimating guide-key occurrences in reads

Given a guide of size *s* bp, a *key* of size *k* bp, a read length of *L* bp, and a ratio *r* of possible guides among all bp combinations of size *s*, the following formula n = (L - n*s - k + 1) * r approximates the number of total guide-keys in a random read. It follows that *n = (L-k+1)/(s+1/r)*. *r =* 12 / 4^3^ for XYX while *r = 4 /* 4^3^ for XXX.

### Algorithm for read mapping and sequence alignments

The algorithm for mapping reads is shown in Supplementary Figure S8. Briefly, if the forward-read is mapped, then intersecting key positions of the reverse-read defines whether both reads are mapped or only the forward one. If the forward-read cannot be mapped, then the reverse-read defines whether both reads are unmapped, only the reverse-read is mapped, or both reads are mapped if intersecting keys are found. To define a read as mapped, first the schedule function searches for candidates that are then quickly compared using the compressed genome without considering insertions and deletions, and set as mapped if equal residues are > = 93.5% (1 - read_length/16); otherwise, a full alignment considering insertions and deletions is performed using the dynamic-programming library WFA2 (https://github.com/smarco/WFA2-lib) and set as mapped if equal residues are > = 87.5% (1 – read_length/8). A summary of how many full alignments were performed is shown in the normal output after mapping.

### Variant calling

To evaluate the downstream effects of ogMapper, we compared the differences in variant calling across aligners. For variant calling, we avoided callers that do not realign reads around variants. Thus, we used GraphTyper2 (Eggertsson et al., 2019). Overall, the prototype command for variant calling was “<aligner to sam> | samtools fixmate -O bam -u | samtools sort --output-fmt-option version=3.0 --output-fmt-option reference=chm13v2.0.fa --write-index -O cram | graphtyper genotype --force_no_copy_reference | bcftools concat --naive”. For comparison across aligners, we used “bcftools view -i 'FORMAT/AD[GT] >= 4 && DP >= 10' -Oz -W=tbi | bcftools isec -p”. For ogMapper, we used the index generated by XYX guider, k=24, and 1-bit AT=0_GC=1 Encoding. We used 232 million 150bpx2 reads, estimating a depth of 22.5X. For the HaplotypeCaller, we used GATK v4.7.0.0 and ran a command like “java -jar ./gatk.jar HaplotypeCaller --native-pair-hmm-threads 16 -R chm13v2.0.fa -I <cram> -O <vcf>”. For mPileUp, we used bcftools v1.21-32 employing a command as ”./bcftools mpileup --threads <threads> -f chm13v2.0.fa -B -Ou -o <output> <cram>”.

## Data Availability

The C++ code is available on GitHub as https://github.com/vtrevino/ogmapper, including installing, compiling, and running instructions for Unix-like operating systems (Linux, Android, Mac OSX). Installation through conda is also available as ‘ogmapper’ package, as shown in the GitHub page. Reads were taken from the sample U0a of the Genome in a Bottle project NA12878:HG001 WGS HighSeq 300x from https://github.com/genome-in-a-bottle/giab_data_indexes, particularly those of the L001 pulled together (U0a_CGATGT_L001_R*_00*.fastq.gz) from https://ftp.ncbi.nlm.nih.gov/ReferenceSamples/giab/data/NA12878/NIST_NA12878_HG001_HiSeq_300x/131219_D00360_005_BH814YADXX/Project_RM8398/Sample_U0a/, which reach 18,203,885 paired-end reads. For the variant calling, we used all available files from the same directory beyond L001 aggregated to 232,318,384 paired reads for a depth estimate of 22.5X. The original contributions presented in the study are included in the article or its supplementary material; further inquiries can be directed to the corresponding author(s). For annotating the variants, we used the files for centromeres (chm13v2.0_censat_v2.1.bed) and exons (chm13v2.0_RefSeq_Liftoff_v5.3.gff.gz) regions from https://github.com/marbl/CHM13. The variants used as benchmark for the HG002 sample from the Genome in a Bottle project were obtained from https://42basepairs.com/browse/web/giab/release/AshkenazimTrio/HG002_NA24385_son/v5.0q around the 9th of September 2026, specifically the files HG002_CHM13v2.0_v5.0q_smvar.benchmark.bed and HG002_CHM13v2.0_v5.0q_smvar.vcf.gz. For mapping the HG002 sample, we used the first 256 paired-end sequence files in https://github.com/genome-in-a-bottle/giab_data_indexes accessed around the 1st of September 2026, specifically the sequence files within “Illumina WGS 2x150bp 300X per individual” from the HG002 sample at https://ftp-trace.ncbi.nlm.nih.gov/ReferenceSamples/giab/data_indexes/AshkenazimTrio/sequence.index.AJtrio_Illumina300X_wgs_07292015_updated.HG002. Read 1 and read 2 files were correspondingly concatenated generating an overall R1 and R2 files of around 92 GiB each containing 847,410,996 paired reads for an approximately 80x of depth coverage.

## References

[B1] Betschart R. O. Thiéry A. Aguilera-Garcia D. Zoche M. Moch H. Twerenbold R. (2022). Comparison of calling pipelines for whole genome sequencing: an empirical study demonstrating the importance of mapping and alignment. Sci. Rep. 12, 21502. 10.1038/s41598-022-26181-3 36513709 PMC9748128

[B2] Canzar S. Salzberg S. L. (2017). Short read mapping: an algorithmic tour. Proc. IEEE 105, 436–458. 10.1109/JPROC.2015.2455551 28502990 PMC5425171

[B3] Danecek P. Bonfield J. K. Liddle J. Marshall J. Ohan V. Pollard M. O. (2021). Twelve years of SAMtools and BCFtools. Gigascience 10, giab008. 10.1093/gigascience/giab008 33590861 PMC7931819

[B4] Davis-Turak J. Courtney S. M. Hazard E. S. Glen W. B. da Silveira W. A. Wesselman T. (2017). Genomics pipelines and data integration: challenges and opportunities in the research setting. Expert Rev. Mol. Diagnostics 17, 225–237. 10.1080/14737159.2017.1282822 28092471 PMC5580401

[B5] Dwarshuis N. Kalra D. McDaniel J. Sanio P. Alvarez Jerez P. Jadhav B. (2024). The GIAB genomic stratifications resource for human reference genomes. Nat. Commun. 15, 9029. 10.1038/s41467-024-53260-y 39424793 PMC11489684

[B6] Edgar R. (2021). Syncmers are more sensitive than minimizers for selecting conserved k-mers in biological sequences. PeerJ 9, e10805. 10.7717/peerj.10805 33604186 PMC7869670

[B7] Eggertsson H. P. Kristmundsdottir S. Beyter D. Jonsson H. Skuladottir A. Hardarson M. T. (2019). GraphTyper2 enables population-scale genotyping of structural variation using pangenome graphs. Nat. Commun. 10, 5402. 10.1038/s41467-019-13341-9 31776332 PMC6881350

[B8] Hansen N. F. Dwarshuis N. Ji H. J. Rhie A. Loucks H. Logsdon G. A. (2026). A complete diploid human genome benchmark for personalized genomics. Cell 189, 4857–4875.e31. 10.1016/j.cell.2026.06.016 42561913 PMC13456413

[B9] Jung Y. Han D. (2022). BWA-MEME: BWA-MEM emulated with a machine learning approach. Bioinformatics 38, 2404–2413. 10.1093/bioinformatics/btac137 35253835

[B10] Langmead B. Salzberg S. L. (2012). Fast gapped-read alignment with bowtie 2. Nat. Methods 9 (4 9), 357–359. 10.1038/nmeth.1923 22388286 PMC3322381

[B11] Li H. (2018). Minimap2: pairwise alignment for nucleotide sequences. Bioinformatics 34, 3094–3100. 10.1093/bioinformatics/bty191 29750242 PMC6137996

[B12] Li H. Homer N. (2026). Fast Genomic Read Alignment with Minibwa. 10.48550/arXiv.2606.15357.

[B13] Md V. Misra S. Li H. Aluru S. (2019). Efficient architecture-aware acceleration of BWA-MEM for multicore systems. Proceedings - 2019 IEEE 33rd International Parallel and Distributed Processing Symposium, IPDPS 2019, 314–324. 10.1109/IPDPS.2019.00041

[B14] Polonis K. Blommel J. H. Hughes A. E. O. Spencer D. Thompson J. A. Schroeder M. C. (2025). Innovations in short-read sequencing technologies and their applications to clinical genomics. Clin. Chem. 71, 97–108. 10.1093/clinchem/hvae173 39749506

[B15] Poplin R. Ruano-Rubio V. DePristo M. A. Fennell T. J. Carneiro M. O. Van der Auwera G. A. (2017). Scaling accurate genetic variant discovery to tens of thousands of samples. 10.1101/201178

[B16] Shaw J. Yu Y. W. (2022). Theory of local k-mer selection with applications to long-read alignment. Bioinformatics 38, 4659–4669. 10.1093/bioinformatics/btab790 36124869 PMC9563685

