# Supplementary material for: ogMapper improves speed and memory for short-sequencing read mapping using a novel guided sparse genome index
Source: Front Bioinform. 2026 Oct 19;6:1912289. doi: 10.3389/fbinf.2026.1912289 (PMC13725094; doi:10.3389/fbinf.2026.1912289)
Supplement: Supplementary file 1 [file Supplementaryfile1.docx]

**Supplementary Information**

# ogMapper improves speed and memory for short sequencing read mapping using a novel guided sparse genome indexing

Victor Treviño^1,*^ and Eugenio Guzman-Cerezo^1^

^1^ Tecnologico de Monterrey, oriGen Project. Monterrey, Nuevo León, 64849 México.

* Corresponding author: vtrevino@tec.mx

## Supplementary Tables

**Supplementary Table 1.** Expected and observed key ratios.

| Guide Pattern | Expected Key-Sequence Ratio | Keys  (T2T v2  N=3.16 Gpb) | Observed Key-Sequence Ratio | Keys expected in 150 nt short- reads |
| --- | --- | --- | --- | --- |
| XYX (Y <> X) | 0.1875 | 448,245,013 | 0.1420 | 12.4 |
| XX | 0.2500 | 707,973,549 | 0.2243 | 17.2 |
| XXX | 0.0625 | 734,111,053 | 0.2326 | 5.4 |
| XXX+ | <0.0625 | 731,166,895 | 0.2317 | <5.4 |
| XXYY (Y <> X) | 0.0469 | 788,355,754 | 0.2498 | 4.2 |
| 4nt Palindrome | 0.0469 | 922,680,201 | 0.2923 | 4.2 |

**Supplementary Table 2.** Encoding DNA Keys.

| Encoding *E()* | Binary Key for  T T C A C C C G | Bit Size** | Parameter recommendation (-k) |
| --- | --- | --- | --- |
| Plain* | 11 11 01 00 01 01 01 10 | 2n | Multiples of 4 |
| Bitwise AT=1, GC=0 | 1 1 0 1 0 0 0 0 | n | Multiples of 8 |
| Swapping AT=1, GC=0 | 1 0 0 0 | n/2 | Multiples of 16 |
| Gapped AT=1, GC=0 | 1 1 0 0 0 0 | 2n/3 | Multiples of 12 |

*** 2-bit packing:** A=00, C=01, G=10, T=11
**** n** = Key size in nucleotides

## Supplementary Figures

**Supplementary Figure 1.** Index building and access. An index is built with all possible combinations of encoded keys. This helps to make direct access from encoded keys to all genome positions where that key was present.

**Supplementary Figure 3. Positions per key. a,** distribution of genome positions per key. For example, there are more than 3 million keys containing 8 genome positions for the XXX+ guiding pattern. **b,** distribution of positions per key depending on key encoding. Key size = 24 bits.

**Supplementary Figure 4. Comparison of key length.** Comparison of different key sizes using BitwiseAT1GC0 encoding that use the same number of bits per nucleotide (using -k parameter) **a,** Impact of key size in total index size. **b,** Impact of key size on mapped reads. **c,** impact of key size on running time. **d,** distributions of genome positions per key.

**Supplementary Figure 5. Estimation of the fraction of shared and exclusive variants within centromeres using GraphTyper2 for U0a sample. a,** ogMapper. **b,** bwa-mem2.

**Supplementary Figure 6. Estimation of the fraction of shared and exclusive variants within centromeres using HaplotypeCaller for U0a sample. a,** ogMapper. **b,** bwa-mem2.

**Supplementary Figure 7. Estimation of the fraction of shared and exclusive variants within centromeres using mPileUp for U0a sample. a,** ogMapper. **b,** bwa-mem2.

**Supplementary Figure 8.** Algorithm for mapping paired reads. For mapping, many functions are provided, which are controlled by the -s (schedule) parameter, see git-hub repository for mapping functions.
